# Acceptance of Digital Leisure for Older Adults: Focusing on the Senior Technology Acceptance Model

**DOI:** 10.1093/geroni/igaf122.4308

**Published:** 2025-12-31

**Authors:** Hyunjoo Lee, Hyeri Shin, Yeomin Han, Donghyun Lee, Young Sun Kim

**Affiliations:** Kyunghee University, Seoul, Republic of Korea; Kyunghee University, Seoul, Republic of Korea; Kyunghee University, Seoul, Republic of Korea; Kyunghee University, Seoul, Republic of Korea; Kyunghee University, Seoul, Republic of Korea

## Abstract

Rapid digitalization is reshaping leisure in later life, creating new opportunities for engagement, learning, and social connection (Nimrod & Adoni, 2012). In Korea, smartphone-based leisure use among adults aged 60+ has risen markedly over the past decade (Ministry of Culture, Sports and Tourism, 2018, 2022). Participation in digital leisure—covering activities such as online communication, streaming, gaming, and virtual learning—has been associated with enhanced quality of life, subjective well-being, and social inclusion (Regalado et al., 2023; Gallistl & Nimrod, 2019). Understanding the factors that shape acceptance is therefore essential for policies and interventions that promote active and healthy aging. This study tested a structural model linking gerontechnology anxiety, multidimensional health, control beliefs, attitudinal beliefs, and intention to participate in digital leisure. Data from 300 community-dwelling adults aged 60+ were analyzed using AMOS. Health positively predicted control beliefs (β=.36, p<.001), attitudinal beliefs (β=.42, p<.001), and intention (β=.18, p<.01). Control beliefs (β=.28, p<.001) and attitudinal beliefs (β=.51, p<.001) significantly predicted intention, with attitudinal beliefs showing the strongest effect. Technology-related anxiety negatively predicted control beliefs (β = −.15, p<.05) but had a small positive association with attitudinal beliefs (β=.11, p<.05), indicating that mild anxiety may coexist with curiosity and perceived benefits. This dual role suggests that while anxiety can reduce confidence, it may also reflect cautious interest. Interventions that reduce functional barriers, build self-efficacy, and harness the motivational aspects of mild anxiety through supportive, hands-on learning could increase older adults’ digital leisure participation, supporting quality of life, psychosocial well-being, and social integration among older adults.

